# Supplementary material for: Learning to integrate parts for whole through correlated neural variability
Source: PLoS Comput Biol. 2024 Sep 3;20(9):e1012401. doi: 10.1371/journal.pcbi.1012401 (PMC11398653; doi:10.1371/journal.pcbi.1012401)
Supplement: S1 Appendix — The derivation of Eqs 8, 9, 11 and 12. (PDF) [file pcbi.1012401.s001.pdf]

S1 Derivation of input moments in the motion direction detection task

To derive Eq. 8-9 and Eq. 11-12, we first calculate the mean and covariance of the stimulus intensity and its rate of change as $T \to \infty$. Given stimulus intensity

$$I(\mathbf{x}, t) = 1 + c\cos(\mathbf{k} \cdot \mathbf{x} - \omega t),$$

and its rate of change

$$\partial_t I(\mathbf{x}, t) = c\omega \sin(\mathbf{k} \cdot \mathbf{x} - \omega t),$$

Their temporal averages are

$$\mathbb{E}[I] = 1, \mathbb{E}[\partial_t I] = 0.$$

The covariance between the intensity is

$$\mathrm{Cov}[I(\mathbf{x}, t), I(\mathbf{x}', t)] = \lim_{T\to\infty} \tfrac{1}{T} c^2 \int_0^T \cos(\mathbf{k} \cdot \mathbf{x} - \omega t)\cos(\mathbf{k} \cdot \mathbf{x}' - \omega t) dt = \tfrac{1}{2} c^2 \cos[\mathbf{k} \cdot (\mathbf{x} - \mathbf{x}')].$$

Similarly, the covariance between the rate of change is

$$\mathrm{Cov}[\partial_t I(\mathbf{x}, t), \partial_t I(\mathbf{x}', t)] = \lim_{T\to\infty} \tfrac{1}{T} c^2\omega^2 \int_0^T \sin(\mathbf{k} \cdot \mathbf{x} - \omega t)\sin(\mathbf{k} \cdot \mathbf{x}' - \omega t) dt = \tfrac{1}{2} c^2\omega^2 \cos[\mathbf{k} \cdot (\mathbf{x} - \mathbf{x}')].$$

Finally, the covariance between the intensity and its rate of change is

$$\mathrm{Cov}[I(\mathbf{x}, t), \partial_t I(\mathbf{x}', t)] = \lim_{T\to\infty} \tfrac{1}{T} c^2\omega \int_0^T \cos(\mathbf{k} \cdot \mathbf{x} - \omega t)\sin(\mathbf{k} \cdot \mathbf{x}' - \omega t) dt = -\tfrac{1}{2} c^2\omega \sin[\mathbf{k} \cdot (\mathbf{x} - \mathbf{x}')].$$

Similarly, we also establish that

$$\mathrm{Cov}[\partial_t I(\mathbf{x}, t), I(\mathbf{x}', t)] = \tfrac{1}{2} c^2\omega \sin[k \cdot (\mathbf{x} - \mathbf{x}')].$$

Recall that given inhomogeneous Poisson spike trains with instantaneous rates $\lambda_i(t)$ their mean and covariance over a finite time window Δt are

$$\mathbb{E}[n_i] = \lambda_i \Delta t$$

and

$$\mathrm{Cov}[n_i, n_j] = \mathrm{Cov}[\lambda_i, \lambda_j]\Delta t^2 + \lambda_i \delta_{ij} \Delta t$$

respectively. Therefore, given the instantaneous firing rate of the intensity detectors and the change detectors

$$\lambda_i(t) = \alpha I(\mathbf{x}_i, t),$$

and

$$\lambda_j(t) = \beta[1 - \partial_t I(\mathbf{x}_j, t)],$$

respectively, we can calculate the mean and covariance of spike count over finite time windows Δt as in Eq. 8-9 and Eq. 11-12.
