# Supplementary material for: Learning to integrate parts for whole through correlated neural variability
Source: PLoS Comput Biol. 2024 Sep 3;20(9):e1012401. doi: 10.1371/journal.pcbi.1012401 (PMC11398653; doi:10.1371/journal.pcbi.1012401)
Supplement: S2 Appendix — The relationship between weight Win, the preferred direction of hidden neurons and their correlation of the response. (PDF) [file pcbi.1012401.s002.pdf]

S2 Analysis of response correlation among hidden neurons

In Fig. 2**c**, we have demonstrated that the correlation between the weights projected from sensory neurons to hidden neurons is highly dependent on the sensory neurons' functions and spatial locations. We are therefore interested in examining the correlation patterns from the perspective of hidden neurons. Fig. S1**a** illustrates the correlation between the weights received by hidden neurons, sorted by their preferred directions. The correlation pattern clearly indicates that neurons with similar preferred directions tend to receive similar weights from sensory neurons. Consequently, the average response correlation between hidden neurons with similar preferred directions is stronger than between neurons with dissimilar preferred directions (Fig. S1**b**). In particular, when the difference in preferred direction exceeds $\pi/2$ radians, the hidden neurons tend to fire independently (Fig. S1**c**).

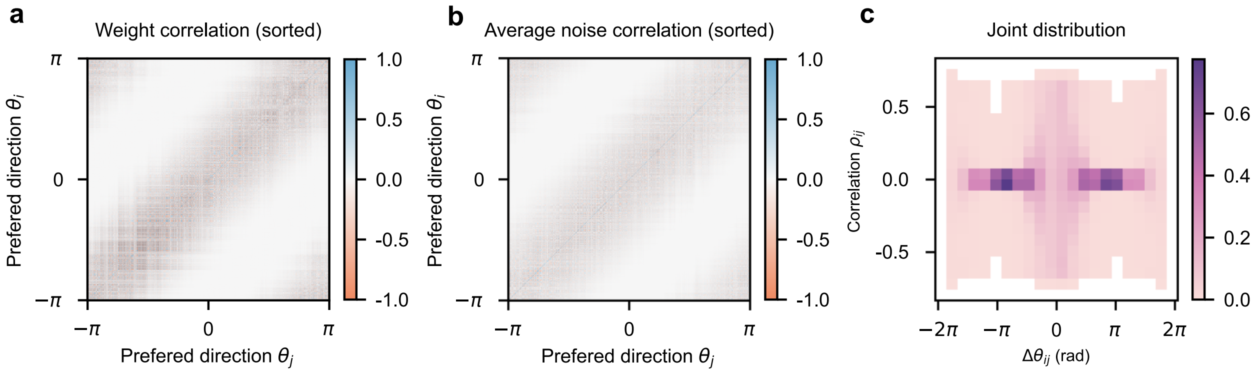

Figure S1: **Analysis of response correlation among the hidden neurons under the high contrast condition. a.** Correlation of the trained connection strengths W_{in} that each hidden neuron receives. The hidden neurons are sorted by their preferred directions. **b.** Average noise correlation among hidden neurons as motion directions vary. **c.** Distribution of noise correlation ρ_{ij} for pairs of hidden neurons $(i \neq j)$ plotted against the difference in their preferred directions ($\Delta\theta_{ij} = \theta_i - \theta_j$. The color bar represents the joint probability density of $\Delta\theta_{ij}$ and ρ_{ij}. In both **a** and **b**, hidden neuron indices are sorted based on their preferred directions, ranging from $-\pi$ to π, and color bars indicate the strength of correlation coefficients. All results are obtained at the high contrast level where $c = 0.8$.
