# Supplementary material for: Learning to integrate parts for whole through correlated neural variability
Source: PLoS Comput Biol. 2024 Sep 3;20(9):e1012401. doi: 10.1371/journal.pcbi.1012401 (PMC11398653; doi:10.1371/journal.pcbi.1012401)
Supplement: S3 Appendix — We conduct a series of experiments to investigate how the gain factors α and β, and the temporal angular frequency ω affect SNN behaviors. (PDF) [file pcbi.1012401.s003.pdf]

S3 Impact of input parameters on the SNN for detecting motion direction

In the motion direction detection task, the instantaneous firing rates of the intensity and change detectors are determined by contrast c, gain factors α and β, and temporal angular frequency ω. In the main text, we have explored the impact of contrast. Here we conduct a series of experiments to investigate how gain factors α and β, and temporal angular frequency ω affect the behaviors of SNN. All experiment settings are the same as in the main text. For simplicity, we consider the case where $\alpha = \beta$.

S3.1 The impact of gain factor

We vary the gain factor from 0.1 to 1.0, maintaining the contrast at $c = 0.8$ and the temporal angular frequency at $\omega = 1$. Under these conditions, the maximum input firing rate now ranges from 180 to 1800 sp/s. Since the model parameters are trained using MNN with the gain factor α fixed at 1, we scale the weight W_{in} by dividing it by α. We find that the gain factor has a minimal impact on model performance. Even with a gain factor significantly below 1, the readout error remains roughly the same and the convergence speed is comparable to that of the default parameters (Fig. S2**a**). In the information-theoretic analysis (Fig. S2**b**), setting the gain factor to 0.1 slows the rate of information decoding, which we argue is a side effect of weight scaling: from a moment mapping perspective, scaling the weight W_{in} by dividing it by α ensures that the mean inputs to hidden neurons match those under the condition $\alpha = 1$, but results in greater variance since the mean and covariance are not scaled equally. In addition, the power spectral density of the normalized population spike count shows little variation between different gain factors, indicating similar patterns of synchronous firing (Fig. S2**c**).

The side effect of weight scaling can be illustrated by hidden neurons' spike trains. As shown in (Fig. S2**d**), hidden neurons whose direction preferences align with the presented stimulus are strongly activated across different gain factors, showing a clear pattern of direction selectivity. The main difference from lowering contrast lies in the spiking behavior of hidden neurons whose direction preferences differ significantly from the presented stimulus; these neurons tend to fire more frequently under low gain factors than high gain factors. This happens because weight scaling amplifies the variance in the input current to hidden neurons, thereby increasing the likelihood that their membrane potentials will surpass the firing threshold.

Overall, we have demonstrated that model performance is not sensitive to variations in the input firing rate or gain factors given appropriate weight scaling.

S3.2 The impact of temporal angular frequency

Our theory suggests that the decomposition of covariance into signal and noise covariance is valid only if the temporal frequency is moderate, neither too fast nor too slow, relative to the observation time window. If the frequency is sufficiently high, the variation in the firing rate within an observation time window will be averaged out. Conversely, if the frequency is too low, the stimuli will resemble static noise, causing the information about the motion direction to diminish. Therefore, we are particularly interested in the range of temporal frequencies in which the model functions correctly.

We test the model with a broad range temporal angular frequencies from 0.2 to 102.4 rad/ms to investigate how the model's behavior is affected. In contrast to the two methods mentioned above to manipulate the input properties, the change in temporal angular frequency ω is more subtle.

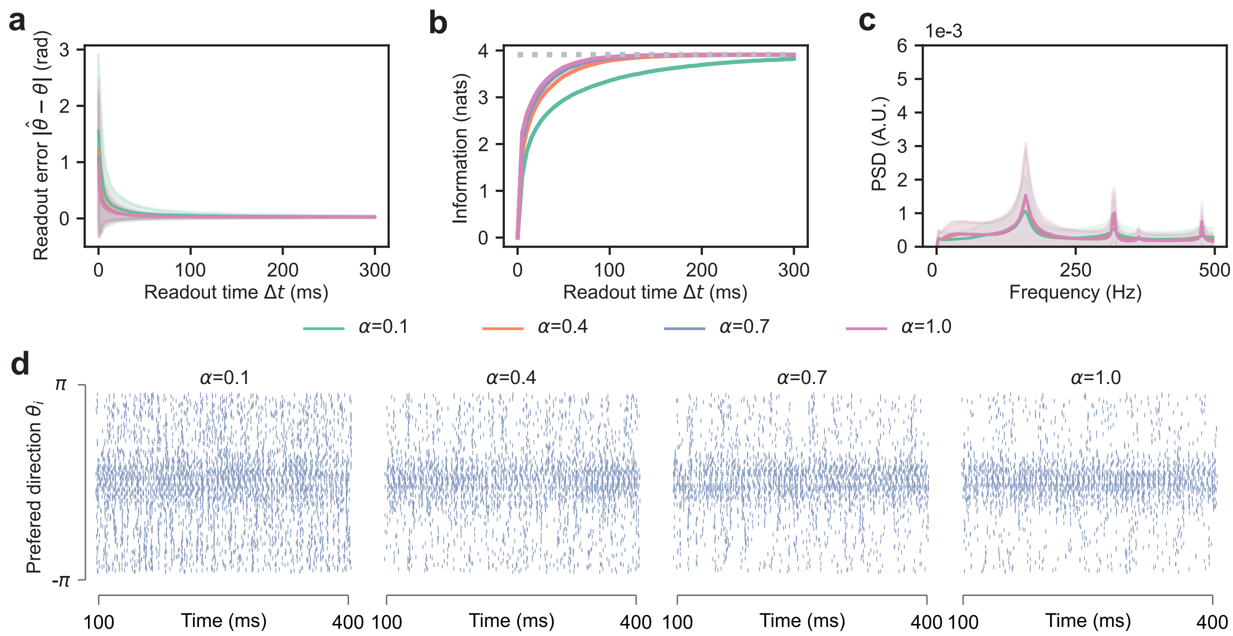

Figure S2: **The impact of gain factors.** **a,** Readout error over time when varying the gain factor. The solid line and shaded region indicate the mean and standard deviation across 500 trials, averaged over 50 motion directions. **b,** Mutual information between the cumulative readout and the motion direction of the presented stimuli under different gain factors. **c,** Power spectrum analysis of the normalized spike count of hidden neurons under different gain factors. **d,** Raster plots of hidden neurons with varying gain factors. The orientation of the presented stimulus is 0.06 radians.

For intensity detectors, ω does not influence their firing rate but only alters the frequency at which the instantaneous firing rate oscillates. For change detectors, ω affects both the amplitude and the frequency of the oscillation. In particular, the change detectors' firing rates can be negative when ω is too high. In these cases, we simply assign a value of -1 to the spikes emitted by the change detectors when their firing rate turns negative. To accommodate high-frequency oscillations, we set the simulation time step to $\delta t = 0.01$ ms for these cases. The contrast and the gain factor are fixed at 0.8 and 1 respectively.

Figure S3 illustrates the readout error at various temporal angular frequencies of the stimulus. We find that the model performs optimally within the frequency range of 0.4 rad/ms to 3.2 rad/ms. Frequencies outside this range, whether higher or lower, degrade performance, resulting in increased readout bias and trial-to-trial variability.

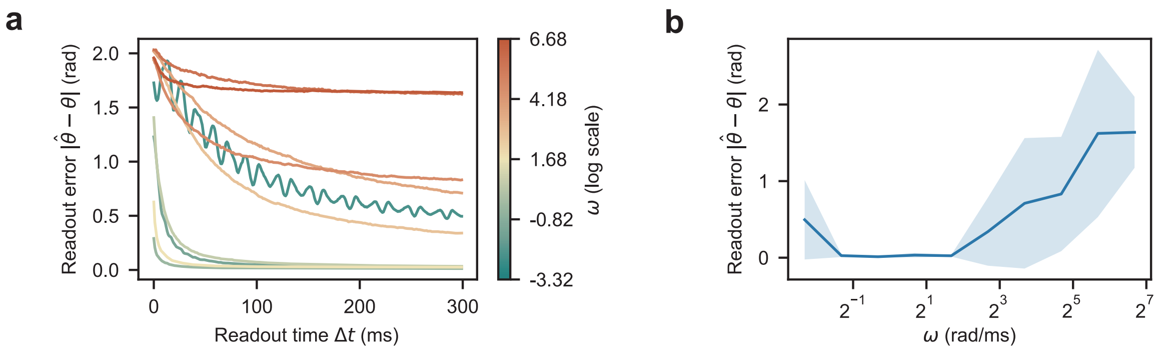

Figure S3: **The impact of gain factors. a,** The mean readout error over time when varying temporal angular frequencies of the inputs. **b,** A comparison of the decoding error under different temporal angular frequencies after 300 ms readout. The solid line represents the mean, and the shaded area shows the standard deviation across 500 trials, averaged over 50 motion directions.
