# Supplementary material for: Learning to integrate parts for whole through correlated neural variability
Source: PLoS Comput Biol. 2024 Sep 3;20(9):e1012401. doi: 10.1371/journal.pcbi.1012401 (PMC11398653; doi:10.1371/journal.pcbi.1012401)
Supplement: S4 Appendix — Comparison of the readout distribution and the Gaussian distribution that have the same mean and covariance in the motion direction detection task. (PDF) [file pcbi.1012401.s004.pdf]

S4 The empirical readout distribution in SNN

In the evaluation of the loss function during MNN training (Eq. 30) and the information-theoretical analysis during SNN inference (Eq. 33 - Eq. 40), we have approximated the readout distribution with a Gaussian distribution. To verify this assumption, we analyze the empirical distribution of the readout against the corresponding Gaussian distribution, utilizing the experimental setup from Fig. 3**a** with varying motion directions. We find that the readouts in the SNN are well captured by a Gaussian distribution, as is consistent with the theoretical argument based on the central limit theorem.

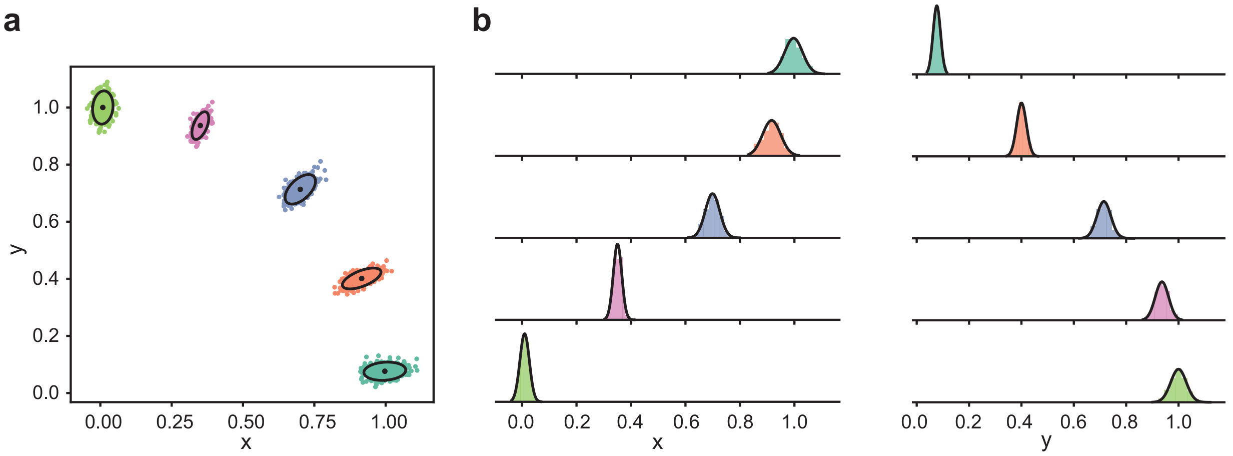

Figure S4: **The empirical readout distribution approximates a Gaussian**. **a,** The joint distribution of readout's x and y coordinates. Colored dots indicate the averaged readouts in one trial under different inputs (500 trials for each direction), while ellipses and black dots show the Gaussian distributions with matching mean and covariance. **b,** The marginal readout distribution. The histogram displays the empirical distribution, and the black curves represent the Gaussian probability density with the same mean and variance.
