# Supplementary material for: Learning to integrate parts for whole through correlated neural variability
Source: PLoS Comput Biol. 2024 Sep 3;20(9):e1012401. doi: 10.1371/journal.pcbi.1012401 (PMC11398653; doi:10.1371/journal.pcbi.1012401)
Supplement: Attachment — Submitted filename: review_plosCB2024.pdf [file pcbi.1012401.s005.pdf]

Review of research paper “Learn to integrate parts for whole through correlated neural variability”

Summary

The manuscript presents several models of neural coding under the common theme of utilizing the covariance of the neural activity to process information. Authors propose an encoding scheme of perceptual information based on covariance of sensory neurons, followed by a decoding scheme where downstream neurons learn to decode covariance-based information and reflect this information in their firing rates. Furthermore, authors show that covariance slightly improves the performance on a classification task with natural images.

General

While the manuscript contains interesting ideas and some compelling results, the paper lacks clarity and consistency and it is unclear what exactly is the main result. While most of individual sentences are clear, the lack of clarity arises from how different parts of the manuscript connect to each other. There seem to be only a loose connection around the hypothesis of utilizing the information in the covariance of the neural activity for coding, or if there is a stronger connection, authors do not explain it clearly. Every section seems to start a new set of results and it is particularly unclear how does the last section of results on a classification task relate to previous parts. Also, there is a problem with how the results are framed, as the introduction and discussion bring in a variety of considerations that are off topic, harming the clarity of the paper. However, seversl results are of interest to the readership of PLOS Computational Biology. For this reason, I suggest a major revision of the manuscript. Unless there is a good reason that the authors can give about how the last section relates with the rest of the paper, I suggest to remove this section, as it does not seem to articulate well with the rest of the paper.

Specific comments, with line number in []

[1-6] The first paragraph of introduction, as well as the title of the paper, presents a part-whole problem. It is unclear in what way this problem is addressed or solved with the results presented in this paper. Further clues on this are given in the 3rd paragraph of introduction. Why not merging these paragraphs together? Even in this case, the title for me does not capture the actual main finding of the paper.

[26] “information grows linearly decodable” is an awkward expression and I am not sure what exactly does it mean.

[38] It is claimed that spiking activities are temporally noisy. The fact that spiking activity is irregular, which is a common observation, does not necessarily imply it is noisy, e.g., that it does not carry information in its timing. Spike timing does not carry the information under the assumption of rate coding. Some models, such as efficient coding with spikes (Boerlin et al. PLOS CB 2013, Koren and Panzeri NeurIPS 2022) instead suggest that spike times are placed precisely at moments when the network updates its internal representation and thus carry information.

[45] Authors mentions on several occasions the perceptual disentangling hypothesis. Could they state more clearly what exactly this hypothesis proposes? Why does perceptual information necessarily need to be reformatted in firing rates of single neurons?

[52-55] This sentence is difficult to read.

[57] Authors mention “synergistic coactivity of neurons”, but it is unclear if they talk about synergy as a defined information-theoretic quantity, or in a looser sense of “neurons coding together”. Many empirical results show that pairs of neurons in sensory areas encode stimuli redundantly.

[84] “new type of role”..why not simply say “new role”?

[109] The variable k should be explained immediately after the Eq.(1)

[127 - 128] Where is this result shown? How does it relate to Eq.(1)?

[31-32] I would expect that sensory neurons convey the information about the stimulus contrast as well.

[141 - 144] Authors make an interesting claim about the neural network shifting the information from covariance to the firing rate, but they do not show it. This is an important claim that should refer to a figure .

[145] It is unclear why another task is examined in this section. Is this necessary, and if so, why?

Eq. 8, 9, 11 and 12: it is not entirely clear how these equations were obtained, could authors put more explanation in Methods?

[186] Authors comment on the problem of stationarity assumption, needed for the model but not compatible with using a non-homogeneous Poisson process. They could show concrete examples about how slow the firing rate must vary and what happens when the variations are too rapid.

[213] The reference to Fig 2 is missing.

Fig 2g: Is it true that higher contrast results in lower firing rates? This seems counterintuitive. Moreover, if the Fano factor and the firing rates have the same profiles, what is the information that is carried by the covariance and it is not carried by the firing rate?

[270] Abbreviation SNN is used but I believe it has not been introduced. Also, it is unclear how the MNN relates to leaky integrate and fire neuron. This seems important and should be explained.

[274] The section number is not visible.

[275] Firing rates of 200 to 1800 spikes peer second seem to be biologically implausible. Why so high?

[paragraph starting 283] The power spectral analysis is unclear.

[paragraph starting 350] The CNN seems to be utilized as a “sensory system for image encoding”, but this network extracts specific complex features from the image such as the beak of the bird. Extracting such complex features seems beyond of what a sensory system in the brain would do.

[408] The comparison between covariance-based information processing and combinatorial code is unclear.

[428] “overall perspective of population activity” is an expression I do not fully understand.
