# Supplementary material for: Learning to integrate parts for whole through correlated neural variability
Source: PLoS Comput Biol. 2024 Sep 3;20(9):e1012401. doi: 10.1371/journal.pcbi.1012401 (PMC11398653; doi:10.1371/journal.pcbi.1012401)
Supplement: Attachment — Submitted filename: Response to reviewers-PLOS CB.pdf [file pcbi.1012401.s006.pdf]

Response to reviewers

General response

We thank the reviewers for the positive comments as well as the thoughtful suggestions and criticisms. In the revised manuscript, we have included additional experiments to address the reviewers' concerns and significantly improved the clarity of the writing.

General changes made to the manuscript are outlines as follows:

- Added new experiments testing the performance of SNN under a range of input conditions. These new results are presented in the Supporting Information.
- Reorganized and rewrote the Abstract, Author Summary, Introduction, and Discussion.
- Thoroughly revised the main text to improve the clarity of writing.
- Improved the layout of some plots to improve visibility.

A couple of changes other than those suggested by the reviewer were made:

- In Fig. 2f, we changed the y-axis from 1/sigma to 1/sigma^2. This reveals that the readout variance is roughly inversely proportioanl to contrast, as consistent with previous study [1] on neural population code.
- In Fig. 3b, we removed the inset plot that shows the readout error during the initial 100 ms and restricted the readout period to 300 ms. This adjustment was made because the readout error stabilizes after 100 ms, making 300 ms sufficient to demonstrate the reduction in readout error over time.
- After careful consideration, we removed the phase-time plot in Fig. 3d. This is because the linear increase of the phase of the membrane potential is rather trivial (e.g. a neuron receiving constant input), and there is no useful conclusion can be drawn from it.
- In Fig. 4a, we replaced the photograph with one taken by us to meet the copyright requirements. The corresponding feature maps and flattened temporal remapping sequences were also replaced.

A point-by-point response to specific concerns raised by the reviewers are listed below.

All changes in the manuscripts have been highlighted.

Response to reviewer #1

> **Q1**: This manuscript by Zhu et al is a very nicely written paper with a potentially powerful idea. They introduce the idea that the covariance structure of the responses of a populations of neurons can be very informative about the identity of a stimulus. It is shown that this covariance structure can be

transformed into a rate code in the output layer of feed-forward spiking networks, and they showed clearly that covariance information can be important in certain cases, as illustrated in the orientation task and the bird categorization task. I would be happy to recommend it for publication once the points described below have appropriately addressed.

-My main comment is that the idea of using the covariance as way to transmit information and transform into rates is very similar to the one developed in the following 3 papers:

The covariance perceptron: A new paradigm for classification and processing of time series in recurrent neuronal networks | PLOS Computational Biology

https://journals.plos.org/ploscompbiol/article?id=10.1371/journal.pcbi.1008127

Covariance-based information processing in reservoir computing systems | bioRxiv

https://www.biorxiv.org/content/10.1101/2021.04.30.441789v2.abstract

Covariance Features Improve Low-Resource Reservoir Computing Performance in Multivariate Time Series Classification | SpringerLink

https://link.springer.com/chapter/10.1007/978-981-16-9573-5_42

In these papers the authors also show that covariance coding can solve some challenging problems, such as audio recognition. The central idea seems to be very close to the one proposed in the paper, so a thorough comparison between the previous work and the current manuscript's goals in the introduction and discussion is needed.

After carefully comparing these previous works listed above, we find that both of these works and ours share similar spirits but also have significant differences. Overall, both these works and ours place a strong emphasis on computing with covariance naturally occurring in neural activity for processing time series data, and significantly differ from conventional deep learning models for processing covariance data, which makes no reference to any underlying stochastic process.

The key difference of the covariance computation considered in our work from that in [2] is that it is modeled after spiking neural networks whose mean firing rate and firing covariability are nonlinearly coupled. As we have demonstrated, this intermingling between moments enables information represented in the firing covariability of upstream neurons to be passed to the mean firing rate of downstream neurons near-losslessly. Our theory based on spiking neurons also has strong biological relevance and can potentially be used to generate quantitative predictions that can be tested against experiments in human and animals.

These previous works have been now properly mentioned in the Introduction (Line 50-65) and a more

throughout comparison has been added to Discussion (Line 423-449).

Q2: Are not spike rates used too high? In Line 275, up to 1800 spikes per second? How the performance of the spiking network degrades with lower rates?

The high input firing rate is primarily due to the gain factor for converting image intensity to firing rates, which is set to 1 sp/ms by default in the original manuscript. To test the performance of our model under more biologically plausible input rates, we have conducted additional simulations with SNN by systematically varying the input conditions:

- Varying the constrast from 0.2 to 0.8.
- Varying the gain factor from 0.1 to 1.
- Varying the angular frequency of the stimulus (corresponding to the speed of motion) from 0.2 to 102.4.

We find that while the contrast signficantly impacts the performance of the SNN, the gain factor does not, indicating that the model performance does not rely on specific input rate.

There new results are presented in the Supporting Information S3.

Q3: In Fig. 4 it is unclear how feature maps are sampled to convert the static bird image into a temporal sequence. Are these features sampled one after the other, and from them the rate is computed, from which means and covariances are finally built.

The feature maps outputed by the pre-trained convolutional layers have the shape (c,h,d) where c = 512 is the number of feature maps (channels) and h=d=28 are the height and width of each feature map, respectively. Each feature map is then flattened to obtaine a temporal sequence of length h*d. The mean and covariance of these sequences, with shapes c and (c,c), are then used as inputs to the MNN classifier. We have clarified this on Line 369-377 and Line 693-698 in the revised manuscript.

Q4: How the analytical expression for Eqs. 24-26 are found? For LIF neurons, expressions for Eq. 24 are well-known for the case of inputs with static mean and covariance, but the expression for the correlation in Eq. 26 can be complicated:

Phys. Rev. Lett. 96, 028101 (2006) - Auto- and Crosscorrelograms for the Spike Response of Leaky Integrate-and-Fire Neurons with Slow Synapses

https://journals.aps.org/prl/abstract/10.1103/PhysRevLett.96.028101

A more detail description of the required methods and comparison between different existing methods needs to be made.

Thanks for pointing out this oversight of missing details. In the revised manuscript, we have included

the definitions of these moment mappings and briefly explained how they are derived in Methods (Section 4.2). Since the maths are quite involved, we have kept this brief to clearly outline the essential ideas and point interested readers to our previous work for full technical details [3], which include a comprehensive comparison to other methods for evaluating the moment mappings.

Currently, the MNN considered in our work is derived based on the assumption of stationary processes, and as a result the correlation considered here is limited to spike count correlation over large windows. Specifically, the connection between the spike count correlation over large time window and spike cross-correlogram is given by Eq. 18 of [4]. How to generalize it to non-stationary process with cross-covariance for different time lags is a challenging open question, both on mathematical and implementation levels. At the mathematical level, the main challenge is the derivation of a self-consistent and closed set of equations involving the cross-covariance. At the implementation level, the main challenge is to develop numerical schemes that can scale up the model to a large network beyond a single pair of neurons.

We have included a paragraph (Line 518-529) to discuss these limitations and challenges in Discussion.

> **Q5**: Is a linear approximation of the output correlation being used? This seems to be only valid, according to the previous literature cited above, when LIF neurons are driven suprathreshold, but not subthreshold.

That is correct. While the moment mappings for the mean and variance are exact up to a diffusion approximation, the correlation mapping is based on linear response theory. The validity of this approximation for the correlation mapping for the LIF neuron model has been documented in detail in our previous work [5]. It is found that the linear approximation works reasonably well for most input regions, even when the input correlation is away from zero. While the approximation does break down for some input range [5], we find that it does not impede task performance, as verified with SNN simulation in the main text.

> **Q6**: The layers described in Lines 502-503 are not very clearly explained. How normalization is performed?

We have included cross-referencing to relevant equations to improve the clarity. For the moment batch normalization, we find that a full explanation with equations involve too much technicality which may distract the reader. Therefore, we have included a more detailed description in words and points interested readers to our previous work for mathematical details [Line 604-610].

> **Q7**: In Line 508, output responses x do not need to be Gaussian, specially if neurons are subthreshold. Why is this approximation good here? Alternatively, one could have sampled x’s from the forward transmission of inputs to the response neurons to get a better generative model for x. Would be any

difference if using the second approach?

The loss function is designed for training the MNN which outputs the mean and covariance of the readout. Given they are the only variables accessible for the loss function the second option isn't possible without full simulation of the SNN (which is computationally expensive), so one has to make some assumptions about the readout distribution. In fact, we argue that the assumption for Gaussian distribution is quite reasonable, as the linear readout involves a weighted sum of a large number of terms such that the central limit theorem applies. This is further verified by comparing the joint and marginal distributions of the readout from SNN simulation with MNN (see Supporting information S4).

Response to Reviewer #2 Dr. Veronika Koren

Q8: General

While the manuscript contains interesting ideas and some compelling results, the paper lacks clarity and consistency and it is unclear what exactly is the main result. While most of individual sentences are clear, the lack of clarity arises from how different parts of the manuscript connect to each other. There seem to be only a loose connection around the hypothesis of utilizing the information in the covariance of the neural activity for coding, or if there is a stronger connection, authors do not explain it clearly**.** Every section seems to start a new set of results and it is particularly unclear how does the last section of results on a classification task relate to previous parts. Also, there is a problem with how the results are framed, as the introduction and discussion bring in a variety of considerations that are off topic, harming the clarity of the paper. However, seversl results are of interest to the readership of PLOS Computational Biology. For this reason, I suggest a major revision of the manuscript. Unless there is a good reason that the authors can give about how the last section relates with the rest of the paper, I suggest to remove this section, as it does not seem to articulate well with the rest of the paper.

Specific comments, with line number in []

[1-6] The first paragraph of introduction, as well as the title of the paper, presents a partwhole problem. It is unclear in what way this problem is addressed or solved with the results presented in this paper. Further clues on this are given in the 3rd paragraph of introduction. Why not merging these paragraphs together? Even in this case, the title for me does not capture the actual main finding of the paper.

We have rewritten the introduction and enhanced the connections between sections to more clearly present our case.

The relationships among the five sections in the Results are as follows:

- Section 1: This section introduces the basic idea of covariance coding and covariance-based

computation with a straightforward example.

- Section 2: This section presents a more specific task to illustrate how covariance can be utilized to encode stimuli.
- Section 3: This section explains how the downstream network decodes motion direction and represents it in the mean firing rate within the MNN framework.
- Section 4: Given the strong connection between MNN and SNN, this section delves into the temporal dynamics of SNN in performing covariance-based computations.
- Section 5: This section extends the experiments to demonstrate that our theory applies not only to simple tasks but also to more complex tasks related to higher cognitive functions in the brain.

While simple stimuli such as the moving grating we used in Section 2-3 are great as a proof of concept, we find it is also improtant to show how our theory is applicable to higher cognitive processing. The effort we made in Section 5 allows us to explore how brain may potentially use neural correlation to handle natural stimuli beyond simple visual processing.

Therefore, we argue that Section 5 has a strong connection to the rest of the paper as clealry pointed out on Line 360-368 and line 530-539 of the revised manuscript.

We consider the title "Learning to integrate parts for whole through correlated neural variability" to be appropriate for this study because:

- The terms "part" and "whole" succinctly encapsulate a central concept of this research, where information is encoded not in single neurons (parts) but in their joint co-fluctuations (whole).
- "Learning to integrate parts for whole" signifies that the aim and outcome of the learning process, which are achieved "through correlated neural variability".

The title should make more sense now given that the main text after revision presents more clearly this idea.

> **Q9**: [26] “information grows linearly decodable” is an awkward expression and I am not sure what exactly does it mean.

We have changed this phrase to "information becomes increasingly linearly separable" (Line 56).

> **Q10**: [38] It is claimed that spiking activities are temporally noisy. The fact that spiking activity is irregular, which is a common observation, does not necessarily imply it is noisy, e.g., that it does not carry information in its timing. Spike timing does not carry the information under the assumption of rate coding. Some models, such as efficient coding with spikes (Boerlin et al. PLOS CB 2013, Koren and Panzeri NeurIPS 2022) instead suggest that spike times are placed precisely at moments when the network updates its internal representation and thus carry information.

We thank the reviewer for raising this interesting point. It can be argued that a "noisy" spike train does not contradict the idea of spike time coding. In fact, our theory of covariance computation is rooted in probabilistic coding, which is somewhat between strict rate coding and spike time coding. According to our theory of covariance computation, information can be represented both in the firing rate, which corresponds to a rate code, and firing covariance, which summarizes certain aspects of the relative timing similar to a spike time code. A key insight is that the representation can be transformed freely between firing rate and covariance through the nonlinear coupling of signal and noise in spiking neurons. Therefore, it can be thought that rate coding and spike time coding are two sides of the same coin. We have added a new paragraph in Discussion (Line 493-505) to clarify these points.

> **Q11**: [45] Authors mentions on several occasions the perceptual disentangling hypothesis. Could they state more clearly what exactly this hypothesis proposes? Why does perceptual information necessarily need to be reformated in firing rates of single neurons?

We have included a paragraph in Discussion (Line 475-492) to clarify the connection between our theory and the perceptual disentangling hypothesis. This hypothesis posits that the objective of information processing in the brain is to render perceptual salience information, which is initially highly entangled in the input space, to become linearly separable.

> **Q12**: [52-55] This sentence is difficult to read.

We have rephrase it to "A growing body of experimental and theoretical works indicate the importance of correlated neural variability in neural population code" (Line 28).

> **Q13**: [57] Authors mention “synergistic coactivity of neurons”, but it is unclear if they talk about synergy as a defined information-theoretic quantity, or in a looser sense of “neurons coding together”. Many empirical results show that pairs of neurons in sensory areas encode stimuli redundantly.

It is meant to refer to synergy in information-theoretic sense. And it is also true that synergy and redundancy can both occur in neural codes, depending on the correlation structure. To clarify, we have rephrased this to: "In particular, through a synergy-redundancy mechanism, neural correlations may enhance or degrade information encoded by a neural population as compared to independent neuronal responses." (Line 29)

> **Q14**: [84] “new type of role”..why not simply say “new role”?

The original intention was to emphasize it's a category of role rather than the specific examples discussed in this paper. But after careful consideration we find that it'd be more informative by being specific, so we have rephrased it to "a more direct role". (Line 87)

Q15: [109] The variable k should be explained immediately after the Eq.(1)

We have rewritten this part to more clearly describe Eq. (1).

Q16: [127 - 128] Where is this result shown? How does it relate to Eq.(1)?

Consider a time series data (in this case, the odor concentration c) decomposed into its Fourier components. If we calculate its average over non-overlapping time bins, as in Eq. (1), then we should expect these averages to reflect the slowly varying components of the data, whereas the fast fluctuations are averaged out. The original statement was intended to refer to this somewhat trivial fact.

However, after reconsideration we find that this fact has low irrelevance to the main idea of the manuscript and therefore have removed it in this revision.

Q17: [131-132] I would expect that sensory neurons convey the information about the stimulus contrast as well.

Thanks for pointing out the oversight. We have removed this errorneous statement.

Q18: [141 - 144] Authors make an interesting claim about the neural network shiftng the information from covariance to the firing rate, but they do not show it. This is an important claim that should refer to a figure .

In the revised manuscript, we have more explicitly explained the transfer of information from correlation to the firing rate using an example with a downstream neuron connected to two sensory neurons, with a new panel in Fig.1b illustrating how the mean firing rates of the downstream neuron changes with the input correlation, even if the mean inputs remain constant.

Q19: [145] It is unclear why another task is examined in this section. Is this necessary, and if so, why?

The binary discrimination task shown in the previous section is a simple example intended mainly for conceptual illustration. The motion direction task is then introduced as a real problem which involves a set of explicit visual stimuli and is solved by training a full network. We have provided additional context at the beginning of this section to indicate its purpose.

Q20: Eq. 8, 9, 11 and 12: it is not entirely clear how these equations were obtained, could authors put more explanation in Methods?

We have included a brief explanation of how these expressions are derived in Supporting information S1.

Q21: [186] Authors comment on the problem of stationarity assumption, needed for the model but not compatible with using a non-homogeneous Poisson process. They could show concrete examples about how slow the firing rate must vary and what happens when the variations are too rapid.

We have included new simulation results for varying oscillation frequency, that is, varying motion speed of the visual grating. These results are presented in Section S3.3 in Supporting Information.

We find that the model performs optimally within the frequency range of 0.4 rad/ms to 3.2 rad/ms. Frequencies outside this range, whether higher or lower, degrade performance, resulting in increased readout bias and trial-to-trial variability.

Q22: [213] The reference to Fig 2 is missing. Fig 2g: Is it true that higher contrast results in lower firing rates? This seems counterintuitive. Moreover, if the Fano factor and the firing rates have the same profiles, what is the information that is carried by the covariance and it is not carried by the firing rate?

We have added the reference to Fig 2a (Line 230).

Fig. 2g actually shows that higher contrast results in higher firing rates. It appears that some confusion is caused by the phrase "Transparency corresponds to contrast levels (higher contrast results in lower transparency)" in the caption. We have changed this phrase to "Opacity corresponds to contrast levels" to remove ambiguity.

Regarding where information is carried: the stimulus-dependent tuning curves are for the hidden neurons, which indeed carry information about motion direction in the firing rate. The information carried by its covariance is not shown, because motion direction is readout from the hidden neuron using linear readout, which does not make use of the covariance. However, the covariance can affect the uncertainty of the readout (i.e. trial-to-trial variability), which is illustrated in Fig. 2e - the covariance ellipse is compressed in the angular direction, implying a reduced uncertainty than uncorrelated readout.

Q23: [270] Abbreviation SNN is used but I believe it has not been introduced. Also, it is unclear how the MNN relates to leaky integrate and fire neuron. This seems important and should be explained.

We have appropriately introduced the term in the introduction (Line 35) and included a brief explanation to how SNN is related to MNN on Line 40-49 and in Methods (see end of Section 4.2).

Q24: [274] The section number is not visible.

We have now enabled numbering for all sections.

Q25: [275] Firing rates of 200 to 1800 spikes peer second seem to be biologically implausible. Why so high?

Please see our response to Q2 by the first reviewer.

The experiments on the impact of firing rates are summarized in Supporting information S3.

Q26: [paragraph starting 283] The power spectral analysis is unclear.

We have more clearly explained the power spectral analysis in the main text on line 312-323 and specified how PSD is calculated in the Method on Line 634-639.

Q27: [paragraph starting 350] The CNN seems to be utilized as a “sensory system for image encoding”, but this network extracts specific complex features from the image such as the beak of the bird. Extracting such complex features seems beyond of what a sensory system in the brain would do.

Thanks for point out this oversight. We are meant to say "... a model for visual processing in early visual cortex" instead of "sensory system". There has been growing interest in using CNNs for modeling visual processing in the brain (see review paper [6]-[7]). While some aspects of CNNs may not be biological, it provides the essential capabilites of feature extraction by the visual cortex.

Q28: [408] The comparison between covariance-based information processing and combinatorial code is unclear.

We have rewritten this paragraph to more clearly explain their connections (Line 450-459).

Q29: [428] “overall perspective of population activity” is an expression I do not fully understand.

The original intention was to emphasize that information carried by a neural population can be different through altering their correlation structure without changing their firing rates. We have rewritten the entire paragraph (Line 450-459) to improve the clarity.

References

[1] Ma, W. J., Beck, J. M., Latham, P. E., & Pouget, A. (2006). Bayesian inference with probabilistic population codes. Nature neuroscience, 9(11), 1432-1438.

[2] Gilson, M., Dahmen, D., Moreno-Bote, R., Insabato, A., & Helias, M. (2020). The covariance perceptron: A new paradigm for classification and processing of time series in recurrent neuronal networks. PLoS computational biology, 16(10), e1008127.

[3] Qi, Y., Zhu, Z., Wei, Y., Cao, L., Wang, Z., Lu, W., & Feng, J. (2023). Toward spike-based stochastic neural computing. arXiv preprint arXiv:2305.13982.

[4] Moreno-Bote, R., & Parga, N. (2006). Auto-and crosscorrelograms for the spike response of leaky integrate-and-fire neurons with slow synapses. Physical review letters, 96(2), 028101.

[5] Qi, Y. (2022). An efficient numerical algorithm for the moment neural activation. arXiv preprint arXiv:2212.01480.

[6] Yamins, D. L., & DiCarlo, J. J. (2016). Using goal-driven deep learning models to understand sensory cortex. Nature neuroscience, 19(3), 356-365.

[7] Lindsay, G. W. (2021). Convolutional neural networks as a model of the visual system: Past, present, and future. Journal of cognitive neuroscience, 33(10), 2017-2031.
