# Supplementary material for: Learning to integrate parts for whole through correlated neural variability
Source: PLoS Comput Biol. 2024 Sep 3;20(9):e1012401. doi: 10.1371/journal.pcbi.1012401 (PMC11398653; doi:10.1371/journal.pcbi.1012401)
Supplement: Attachment — Submitted filename: response_0806.pdf [file pcbi.1012401.s007.pdf]

Response to reviewers

Response to Reviewer #2 Dr. Veronika Koren

> The authors have addressed my questions thoroughly and have made a number of revisions to the text that substantially improved the clarity of the paper. The main results are now clearly presented and important ideas are suitably outlined. While some open questions remain, for example about the biological plausibility of the operating regime of the network (the firing rates of single neurons in the brain are of the order of 1-100 Hz, but in the model they seem much higher), the paper provides a number of results and concepts that will likely be useful for further study of neural coding in biological and artificial networks.
>
> I suggest the Fig. S2 to be placed in the Results section of the main part of the paper, as it provides a comprehensive analysis of the effect of the stimulus contrast on the performance and activity of the network.

Dear Dr. Veronika Koren, we are pleased to acknowledge your suggestion. Appendix S3.1 has been relocated to Sect. 2.4 in the main text (lines 358-383), while the experiments on gain factor and temporal angular frequency have been retained in Appendix S3. In addition, we also corrected some typos.
